# Pediatric Primary Adrenal Insufficiency: A 21-year Single Center Experience

**DOI:** 10.4274/jcrpe.galenos.2020.2020.0132

**Published:** 2021-02-26

**Authors:** Emine Çamtosun, İsmail Dündar, Ayşehan Akıncı, Leman Kayaş, Nurdan Çiftçi

**Affiliations:** 1 İnönü University Faculty of Medicine, Department of Pediatric Endocrinology, Malatya, Turkey; 2 Malatya Training and Research Hospital, Clinic of Pediatric Endocrinology, Malatya, Turkey

**Keywords:** Primary adrenal insufficiency, pediatric, etiology

## Abstract

**Objective:**

Primary adrenal insufficiency (PAI) is a rare but potentially life-threatening condition. In childhood, PAI is usually caused by monogenic diseases. Although congenital adrenal hyperplasia (CAH) is the most common cause of childhood PAI, numerous non-CAH genetic causes have also been identified.

**Methods:**

Patients aged 0-18 years and diagnosed with PAI between 1998 and 2019 in a tertiary care hospital were retrospectively evaluated. After the etiologic distribution was determined, non-CAH PAI patients were evaluated in detail.

**Results:**

Seventy-three PAI patients were identified. The most common etiology was CAH (69.9%, n=51). Non-CAH etiologies accounted for 30.1% (n=22) and included adrenoleukodystrophy (ALD; n=8), familial glucocorticoid deficiency (n=3), Triple A syndrome (n=5), autoimmune adrenalitis (n=1), adrenal hypoplasia congenital (n=1), IMAGe syndrome (n=1), and other unknown etiologies (n=3). The median age at the time of AI diagnosis for non-CAH etiologies was 3.52 (0.03-15.17) years. The most frequent symptoms/clinical findings at onset were hyperpigmentation of skin (81.8%), symptoms of hypoglycemia (40.9%), and weakness/fatigue (31.8%). Hypoglycemia (50.0%), hyponatremia (36.4%) and hyperkalemia (22.7%) were prominent biochemical findings. Diagnosis of specific etiologies were proven genetically in 13 of 22 patients. A novel p.Q301* hemizygous frameshift mutation of the *DAX1* gene was identified in one patient.

**Conclusion:**

Etiology was determined in 86.3% of children with non-CAH PAI through specific clinical and laboratory findings with/without molecular analysis of candidate genes. ALD was the most common etiology. Currently, advanced molecular analysis can be utilized to establish a specific genetic diagnosis for PAI in patients who have no specific diagnostic features.

**What is already known on this topic?**

Primary adrenal insufficiency (PAI) is characterized by deficient production of glucocorticoids and/or mineralocorticoids from the adrenal glands due to dysfunctional or unresponsive adrenal tissue. Congenital adrenal hyperplasia (CAH) is the most common and well-known etiology in childhood. Non-CAH etiologies are rare, and have varying rates of distribution across different populations. There is limited epidemiological and clinical information regarding non-CAH PAI.

**What this study adds?**

To the best of our knowledge, this is the first cohort study of PAI in children from Turkey. We were able to determine the etiology in 95.8% of PAI patients. Non-CAH etiologies accounted for 30% of PAI and are presented in detail, along with a literature review. The most common non-CAH etiology was adrenoleukodystrophy. A potential novel p.Q301* hemizygous frameshift mutation of the *DAX-1* gene was also identified in one patient.

## Introduction

Adrenal insufficiency (AI) is a life-threatening condition characterized by deficient production of glucocorticoids (GC) and/or mineralocorticoids (MC) from the adrenal glands or reduced response to these steroids. If AI is caused by dysfunctional or unresponsive adrenocortical tissue, it is classified as primary AI (PAI). However, if it is caused by disordered function of the pituitary gland and/or hypothalamus, it is termed secondary AI (1). A diagnosis of PAI depends on low serum cortisol and high plasma adrenocorticotropic hormone (ACTH) as well as clinical findings, such as hyperpigmentation of the skin, hypoglycemia, salt wasting, hypotension, and other non-specific symptoms such as fatigue, weight loss, failure to thrive, depression, and convulsions (2,3).

Primary AI has a prevalence of 93-140 per million and an incidence of 4.7-6.2 per million in the white adult populations (4). It is thought to be less common in the pediatric population. In childhood, PAI is usually caused by hereditary or sporadic monogenic disease. Congenital adrenal hyperplasia (70-85%) is the most common cause, with an estimated prevalence of 1/10,000-18,000 (1,2,5,6). In several studies non-CAH etiologies generally accounted for 10-30% of childhood PAI and autoimmune AI was usually the most common (1,5,6). Other genetic etiologies of non-CAH are adrenal gland developmental disorders [X-linked adrenal hypoplasia congenital (AHC), steroidogenic factor-1 related and other syndromic causes], ACTH resistance including familial GC deficiency (FGD) and related conditions and Triple A syndrome (TAS), metabolic causes [cholesterol synthesis/metabolism defects, adrenoleukodystrophy (ALD), other defects of the peroxisome, lysosome, endoplasmic reticulum and mitochondria], GC resistance and aldosterone synthesis/action defects (7,8). Infections, infiltrative diseases, adrenal hemorrhage, bilateral adrenalectomy and some drugs are the non-genetic causes of PAI.

Non-CAH PAI cases are less common, so the exact frequencies of non-CAH etiologies are still unknown, with the exception of ALD which has an estimated prevalence of 1/17000 at birth and the literature contains limited clinical data regarding these rare subgroups (2,8,9). Sharing clinical information about these patients will raise awareness about the disease. Early diagnosis and appropriate treatment is essential for avoiding lethal outcomes in PAI patients.

The aim of this study was to review the etiologies, clinical presentations, laboratory findings, genetic analysis, treatments and follow up features of non-CAH PAI cases that were followed in a pediatric endocrinology department of a tertiary care hospital over a period of 21 years.

## Methods

We retrospectively evaluated patients aged 0-18 years who had their diagnosis and follow up for PAI, between August 1998 and October 2019, at İnönü University Faculty of Medicine, Turgut Özal Medical Center, Department of Pediatric Endocrinology. After the etiologic distribution was determined, non-CAH PAI patients were evaluated in detail. Data was retrospectively extracted from patient records including date of birth, age at diagnosis, sex, clinical characteristics, comorbidities, laboratory results [serum glucose, electrolytes, plasma ACTH, serum cortisol, plasma renin, aldosterone, plasma very long chain fatty acids (pVLCFA), autoantibodies], imaging results (adrenal, central nervous system and other), mutational analysis results, AI etiologies and treatment information. All the information was obtained from clinical records and the hospital’s electronic database, and reviewed by an endocrinologist. Written informed consent forms were filled out by the patients and/or their families so that medical data (including genetic analysis results) may be collected and reported for educational and/or scientific purposes.

Primary AI was diagnosed based on the coexistence of at least the first two of the following criteria: 1) clinical symptoms/signs were suggestive of PAI (recurrent hypoglycemia, hyperpigmentation of skin, hyponatremia with hyperkalemia); 2) plasma ACTH levels at 8 am being twice the upper limit of normal and a cortisol level of <138 nmol/L. If a patient had clinical symptoms and signs suggestive of PAI, but had a serum cortisol level >138 nmol/L at 8 am with high ACTH, a standard dose synacthen stimulation test was performed. Serum cortisol was recorded at 0, 30 and 60 minutes after 250 µg/m^2^ intravenous ACTH. If the peak plasma cortisol level was under 500 nmol/L the patient was also diagnosed with PAI (10); 3) a positive genetic analysis report indicated one of the etiologies of PAI. After PAI diagnosis, CAH subtypes were evaluated by clinical and biochemical analysis initially, and a target gene sequence analysis was done in patients diagnosed with CAH. Only classical CAH patients were included in the study and non-classical cases were excluded. Non-CAH patients were then evaluated for autoimmune adrenalitis and ALD. The presence of 21-hydroxylase-antibody in serum was evaluated by enzyme immunoassay. Plasma VLCFA (pVLCFA) were analyzed with gas chromatography-mass spectrometry. Alacrima was confirmed by using Schirmer’s test. Achalasia was diagnosed based on clinical symptoms and timed barium esophagogram. Brain magnetic resonance imaging (MRI) was conducted in patients who had neurological symptoms or high pVLCFA levels. If certain genetic tests were available, with the permission of the parents, specific genetic analysis was done for target genes in PAI patients. Patients with PAI, who had high pVLCFA levels with/without neurological symptoms/leukodystrophy on brain MRI or a family history of ALD, were diagnosed as ALD clinically and DNA sequencing analysis of the *ABCD1 *gene was done. In patients with PAI, who were also diagnosed with alacrima and/or achalasia, a DNA sequencing analysis of the *AAAS* gene was done to investigate TAS. Patients who had evident growth retardation and other dysmorphic features were evaluated for syndromic PAI etiologies. Patients with early onset PAI who had no specific clinical features were evaluated for *MC2R* and/or *DAX1* gene mutations using DNA sequencing. The genetic analyses were conducted in several different commercial genetic laboratories in Turkey (Detagen, Intergen and Düzen Genetic Laboratories).

This study was approved by the Ethical Committee of İnönü University (approval number: 2019/407), and was conducted in accordance with the World Medical Association Declaration of Helsinki.

### Statistical Analysis

Data were analyzed by descriptive statistical methods. Qualitative variables were expressed as number (%). Continuous quantitative variables were expressed as mean and standard deviation if they conformed to a normal distribution and as median and range if they did not.

## Results

Seventy-three patients were diagnosed with PAI in either an inpatient or outpatient setting over a 21-year period.

### 1. Congenital Adrenal Hyperplasia

CAH was the most common etiology and 51 (69.9%) patients had CAH. Twenty-four CAH patient had 21-hydroxylase deficiency (21-OHD) (47% of CAH), 19 patients had 11-beta-hydroxylase deficiency (11-OHD) (37.2% of CAH), six patients had 17-alpha-hydroxylase deficiency, one patient had 21-22 desmolase deficiency, one patient had steroidogenic acute regulatory protein deficiency. The CAH patients are not reviewed further.

### 2. Non-CAH Etiologies

Non-CAH etiologies accounted for 30.1% (n=22) of PAI: ALD n=8 (11%); FGD n=3 (4.1%); TAS n=5 (6.8%); and autoimmune adrenalitis in one patient, AHC in one patient and IMAGe syndrome in one patient. Etiology of PAI could not be clarified in three patients (4.1%) in whom CAH was excluded (Table 1). Median age at the diagnosis of AI was 3.52 (0.03-15.17) years and male/female ratio was 6.33 (19/3) for non-CAH patients (Table 2). Mean height SDS and weight SDS were normal on admission. The most frequent symptoms and clinical findings were hyperpigmentation of skin (81.8%), symptoms of hypoglycemia (40.9%) and weakness/fatigue (31.8%). The other symptoms and clinical findings were alacrima, adrenal crisis, achalasia, neonatal prolonged jaundice, learning disability, vomiting, headache, low school performance, mental retardation, walking disability, epilepsy, and polyneuropathy. Parental consanguinity was very frequent, and was present in 77.8% (14/18) of cases. Hypoglycemia (50.0%), hyponatremia (36.4%) and hyperkalemia (22.7%) were prominent biochemical findings. Mean plasma ACTH level was very high, while mean serum cortisol level was very low (Table 2). All non-CAH PAI patients were treated with oral hydrocortisone (HC); however, 27.3% of them also received oral fludrocortisone (FC) treatment. The mean follow up duration was 65.04±36.23 months.

### Adrenoleukodystrophy

Eight male patients from four different families were diagnosed with ALD (Table 3). Patient 1 (P1) and P2 were brothers and their family history revealed that four of their brothers had died from AI. P3 was a nephew, who had died of encephalitis when he was seven years old, and P4 was a distant relative of them. P5 and P6 were also brothers from a different family. The other two patients (P7, P8) were from different unrelated families.

Median age at AI diagnosis was 7.17 (2.89-15.17) years for ALD patients. All but one of them presented with hyperpigmentation of the skin. Other symptoms/findings were adrenal crisis, headache, vomiting and hypoglycemia symptoms. In laboratory analysis three patients had hyponatremia and two of them also had hyperkalemia. All patients had low serum cortisol (mean level 105.71±66.79 nmol/L) and very high plasma ACTH levels. Four patients presented with neurological problems (Table 3). Plasma VLCFA levels were evaluated in four patients, and revealed high plasma C26 levels in three and high C26/C22 ratio in all of them. Four patients showed signs of white matter involvement on brain MRI, and one demonstrated a thin corpus callosum and hydrocephalus. Four patients were diagnosed based on clinical and laboratory findings as well as molecular analysis; they all had the same p. P543L (c.C1628T) mutation in the *ABCD1* gene. The other four patients’ diagnoses were based on clinical and laboratory findings with/without family history. All patients were given HC at a median dose of 17.5 mg/m^2^/day. Three patients had MC deficiency (P1 and P3 had mild hyponatremia and high plasma renin activity, P2 presented with severe hyponatremia and hyperkalemia), and needed FC at a dose of 0.05 mg/day, orally. One patient (P4) also had transient hyponatremia and high renin level but he did not need MC replacement. Three patients were prescribed Lorenzo’s oil and lovastatin.

### Triple A (Allgrove) Syndrome

Five patients (P9-13) (three males, two females) from three different families were diagnosed with TAS. P9 and P10 were siblings and P11 was their cousin, the other two patients were from different families without a consanguineous marriage. All patients were living in the same city in east Turkey. Three patients had a classical triad of achalasia, alacrima, AI; the other two had two symptoms from the triad (alacrima and AI). Four of the patients presented with hypoglycemia symptoms and convulsions, all patients had hyperpigmentation of skin, two patients had mild facial dysmorphism, (wide/depressed nasal root, down slanting palpebral fissures, short philtrum), one had mild mental retardation, nasal speech, thenar atrophy and short stature, and the other one had normocytic anemia and corpus callosum hypoplasia. The earliest symptom was alacrima for all patients, which was noticed in early infancy. Hyperpigmentation of skin was noticed at three-five years old. The median age at diagnosis of AI was 6 (3.19-7.83) years old. Transient hyponatremia was seen in one patient; hyperkalemia was not seen. Median serum cortisol level was 30.36 (13.8-218.04) nmol/L, plasma ACTH level was 275 (186.34-440) pmol/L. Molecular analysis of patients revealed the same homozygous mutation p.L356Vfs*8 (c.1066_1067delCT) in the *AAAS* gene. According to the Clinvar database, this mutation (variation ID: 264992) was a previously reported pathogenic mutation (13). All patients were given HC at a median dose of 15 mg/m^2^/day orally, but none of them needed FC. Surgery for achalasia was undertaken for P9 and P10. All patients were prescribed eye drops to prevent dry eyes.

### Familial Glucocorticoid Deficiency

Three patients (P14-16) (two males, one female) from three different families were diagnosed with FGD with genetic confirmation. The age at onset of AI was 1.5, 21 and 2.5 months old, respectively. Two of them presented with hyperpigmentation of skin and hypoglycemia. One patient (P14) also had pes equinovarus and posterior embriyotoxone of the eyes. All of them had a history of prolonged jaundice. Laboratory analysis revealed very low cortisol levels, very high plasma ACTH levels and normal plasma VLCFA levels for all patients. One patient had transient hyperthyrotropinemia. MRI of the adrenal glands was normal in all patients. Genetic analysis showed; P14 and P15 had a homozygous p.G116V (c.347 G>T) mutation in the* MC2R* gene; and P16 had a homozygous p.L225R (c.674T>G) mutation in the *MC2R* gene. All patients were given oral HC at a median dose of 12.5 mg/m^2^/d, but none of them needed FC.

### Congenital Adrenal Hypoplasia

**Figure 1:**
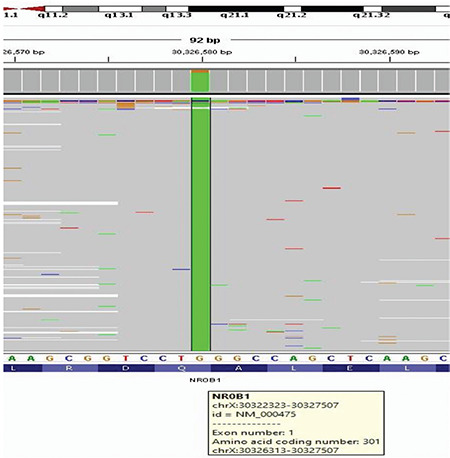
DNA sequence analysis of the patient with adrenal hypoplasia congenital

A twenty-one months old boy (P17), diagnosed with PAI in another medical center at 16 days old was admitted to our hospital. He had salt wasting during AI onset. Medical history revealed that his brother had died due to AI at 10 months old. Biochemical results had shown that he had hyponatremia and hyperkalemia in the neonatal period. At 16 days old his serum cortisol level was low, plasma ACTH level was high, and serum gonadotropin levels were normal. CAH was excluded. He had normal external genitalia. MRI of the adrenal glands was normal. Brain MRI (T2A) showed bilateral hyperintense changes in occipital white matter. Genetic analysis through DNA sequencing revealed a p.Q301* (c.901C>T) hemizygous non-sense mutation in the *DAX1* gene (Figure 1). This variation has not been reported previously; however, it is predicted to result in a premature stop codon and was most likely pathogenic according to both Mutation Taster and ClinVar data (14,15). Genetic analysis of the mother was not performed. He was treated with HC 15 mg/m^2^/day and FC 0.05 mg/day orally.

### Isolated Autoimmune Adrenal Insufficiency

A thirteen-year old male patient (P18) presented with hyperpigmentation of skin, weakness and fatigue. He had no neurological signs. Laboratory analysis showed severe hyponatremia (Na=117 meq/L) and mild hyperkalemia (K=5.5 meq/L). Serum glucose was normal. Serum cortisol level was low, and plasma ACTH level was high. He was examined for autoimmune diseases; thyroid autoantibodies, and celiac antibodies were not detected. Plasma VLCFA test revealed a normal C26 level and high C26/C22 ratio. Mutational analysis of the *ABCD1* gene was normal. In his follow-up, serum 21-hydroxylase antibody was positive and he was diagnosed with isolated autoimmune AI. Left renal agenesis was also revealed during the evaluations. He was treated with HC 10-15 mg/m^2^/day. In the follow up period (47 months), he became obese and demonstrated insulin resistance, type 2 diabetes mellitus, hypertension, hepatosteatosis and dyslipidemia but did not develop any other autoimmune disease.

### Syndromic Primary Adrenal Insufficiency

One male patient (P19) was admitted to another center with adrenal crisis when he was 10 days old. He was diagnosed with PAI, treated with HC and FC, and subsequently referred to our hospital after one month of hospitalization in an intensive care unit. He was born at 41 weeks of gestation with a weight of 2400 g (small for gestational age). He had facial dysmorphic features (frontal bossing, deeply located eyes, hypertelorism, depressed nasal bridge), bilateral undescended testicles, and penile hypospadias. His karyotype was 46,XY, SRY locus (+). His testes exhibited normal hormonal functions. Diagnosis of CAH was excluded. Echocardiogram showed thin patent ductus arteriosus. Abdominal US revealed ectopic and fusioned kidneys. Brain and lumbar MRI revealed multiple hemorrhagic lesions in the brain and a 35x4 mm syringohydromyelia at the lumbar region. It was considered that he may have syndromic PAI due to IMAGe (intrauterine growth retardation-metaphyseal dysplasia-AHC-genital anomalies) syndrome, but genetic analysis has not been performed at the time of writing.

### Unknown Etiology

A total of three male patients were diagnosed non-CAH PAI of unknown etiology.

One male patient (P20) presented with hyperpigmentation and an incidental mass in the right adrenal gland at 13.7 years old. He also had hereditary spherocytosis. He had no neurological/psychiatric complaints or findings. Other than hypoglycemia, biochemical analysis was normal and hormonal assays revealed only partial PAI. Basal level of serum cortisol was normal, but plasma ACTH levels were very high. A standard dose synacthen test resulted in a peak serum cortisol level of 331.08 nmol/L (low). He had no MC deficiency. Plasma VLCFA levels were normal. Adrenal antibodies could not be analyzed, but thyroid autoantibodies and tissue transglutaminase antibodies were negative. Adrenal MRI showed a 4.3x3.1 cm mass in the right adrenal gland. Following right adrenalectomy, the lesion pathology was revealed to be an adrenal hemorrhage. He was given HC replacement.

A two-year-old boy (P21) presented with weakness, hyperpigmentation of the skin, hypoglycemic symptoms including hypoglycemic convulsions. He had no other neurological symptoms or signs. Laboratory analysis showed apparent AI and normal plasma VLCFA levels. Adrenal antibodies were negative. Molecular analysis showed no mutation in the *DAX1* gene. He was treated with HC. The patient was thought to have FGD; however, molecular analysis has not been performed.

A seven-month old boy (P22) presented with hypoglycemia symptoms and hyperpigmentation of the skin. Medical history revealed that he was term with normal birthweight, he had adrenal crisis and hypoglycemic episodes in the newborn period and laboratory analysis showed hyponatremia, hyperkalemia, hypoglycemia, low serum dehydroepiandrosterone sulfate, and very high plasma ACTH. He was treated with HC and FC in another medical center. Physical examination in our hospital revealed that he had microcephaly, diplopia and motor mental retardation. Plasma VLCFA analysis showed a high C26 level, but C26/C22 ratio was normal. His brain MRI revealed cerebral atrophy, calcification in the basal ganglia, and corpus callosum atrophy. Further genetic analysis could not be performed. He was treated with HC and FC for two years.

## Discussion

Primary AI in childhood is a relatively rare but potentially life-threatening condition. Although PAI is mostly caused by monogenic diseases in children, it is often acquired in adults (2).

We reviewed 73 children with PAI over a period of 21 years in a single tertiary center in Turkey. Non-CAH PAI patients were especially reviewed in detail and compared with the literature. To the best of our knowledge this is the first cohort of PAI in children from Turkey. In a previously conducted nationwide cohort study, Guran et al (16) reported clinical and molecular genetic characteristics of children with PAI of unknown etiology (patients with CAH, ALD, autoimmune AI or obvious syndromic PAI such as TAS were excluded) from Turkey.

We were able to determine the etiology in 95.8% (70/73) of all PAI patients and in 86.3% (19/22) of the non-CAH PAI patients in our cohort through clinical and laboratory findings, with some being confirmed genetically.

Although CAH is still the most common cause of childhood PAI at present, numerous non-CAH genetic causes have been identified in the last 25 years, but their prevalance in children with PAI is not yet clear (1,2,5,6). Among CAH etiologies, 21-OHD is the most common (90-95%) (5,6,17). In our cohort, CAH was also the most frequent etiology (69.9%) and 21-OHD was the most common type of CAH (47%). However, in contrast to the literature, 11-OHD (19 cases from 15 families) (37.2%) was also very common in our study. Diagnosis of 11-OHD was confirmed with genetic analysis in 18 of the cases. Racial characteristics and frequent consanguineous marriage in our region might have caused this difference. In a review of 273 Turkish patients with CAH, Kandemir and Yordam (18) reported that 11-OHD was the second most common cause of CAH and accounted for 13.5% of cases, a rate which was still high compared to other populations in which it is reported to be 5-8%.

Similar to the literature, non-CAH causes accounted for 30.1% of childhood PAI in our cohort (Table 1). In contrast, Hsieh and White (3) reported a higher rate (54.5%) of non-CAH etiologies within 77 pediatric PAI patients. Many studies from western countries reported that autoimmune etiologies were the most common cause and accounted for 30-55% of non-CAH PAI in children (1,3,5,11,12). In their Chinese cohort, Wijaya et al (6) reported that ALD (44.9%) and AHC (40.8%) were the most common etiologies in the non-CAH group while autoimmune etiologies only accounted for 6.1%. In our cohort, ALD was the most common etiology (36.3%, n=8), and autoimmune AI was rare (only one patient) which is similar to the study of Wijaya et al (6). This suggests that racial features affect the etiological distribution of AI.

Autoimmune PAI can be isolated or a component of autoimmune polyendocrine syndromes (APS). Detecting anti-adrenal antibodies in serum of patients with PAI leads to the diagnosis of autoimmune PAI. Mutations in the autoimmune regulator gen (*AIRE*), are responsible for APS-1 in which PAI is usually combined with hypoparathyroidism and mucocutaneus candidiasis. APS-2 typically combines PAI with autoimmune thyroid disorders and type 1 diabetes mellitus, and shows a complex inheritance pattern (HLA-DR3/DR4, CTLA-4) similar to isolated autoimmune PAI (2,7). P18, a male adolescent patient had positive serum anti 21-OH antibodies but he had no family history of autoimmunity nor other accompanying autoimmune disease over nearly four years of follow up, and so was ultimately diagnosed with isolated autoimmune PAI.

ALD is an X-linked hereditary metabolic disorder caused by mutations of the *ABCD1* gene, which encodes a peroxisomal transport protein necessary for VLCFA degradation (≥C22). Toxic accumulation of VLCFA in plasma and multiple tissues (white matter of the brain, spinal cord and adrenal cortex) is associated with a proinflammatory state and eventual cell death (19). In male patients, Addison only, cerebral ALD (CALD; childhood, adolescent, or adult onset), and adrenomyeloneuropathy phenotypes can be seen (9). Perry et al (5) reported four ALD patients in their study (two Addison only, one childhood and one adolescent CALD). In our cohort, three patients had Addison only phenotypes, three had childhood CALD, and two had adolescent CALD. Hyperpigmentation of skin was the most common symptom. All patients had either neurological symptoms/white matter involvement, elevated plasma VLCFA, or family history of ALD. ALD causes GC deficiency, and MC deficiency may also be detected. Three of eight ALD patients in our cohort needed MC treatment. Perry reported MC deficiency in one of four ALD patients, while Wijaya et al (6) reported zero cases of MC deficiency in 22 ALD patients (5). If ALD is suspected in a male with neurological symptoms (with or without typical brain MRI abnormalities) or Addison’s disease, diagnosis is made based on elevated VLCFA levels in plasma; genetic confirmation is useful for genetic counselling (9). For molecular confirmation of ALD, a sequence analysis of the *ABCD1 *gene is first performed, and if no pathogenic variant is found, it is followed by a gene-targeted deletion/duplication analysis. This is because the sequence analysis method has a reported diagnostic value of 97% when identifying mutations of the *ABCD1* gene, and the remaining 3% can be detected using deletion/duplication analysis by way of the multiplex ligation-dependent probe amplification method (20). In addition, mutations in regions, such as the promoter region, that play a role in regulating gene expression, may not fall within the sequenced region in sequencing analysis. Four of our patients had a p.P543L mutation in exon 6 of the *ABCD1* gene, which has been reported previously (21) while in two cases *ABCD1 *sequencing analysis revealed no mutation, but subsequent deletion/duplication analysis was not performed. Genotype-phenotype correlation or the trigger for cerebral disease has not been described. The only currently available standard therapy is hematopoietic stem cell transplantation, which should be performed in the early stage of demyelination in boys with CALD.

Triple A (Allgrove) syndrome (OMIM 231550), characterized by achalasia, alacrima, and AI as well as neurological (central, peripheral and autonomic nervous system) and dermatological problems, is caused by homozygous or compound heterozygous mutations in the gene encoding aladin (*AAAS*) on chromosome 12q13. Palmoplantar hyperkeratosis (PH), hypothenar atrophy, short stature, facial dysmorphism, deafness, mental retardation, and nasal speech can also be present (22,23). Even with the same *AAAS* gene mutation, patients have phenotypical heterogenity (23,24). Alacrima was the earliest and the most consistent finding in our five TAS patients; hyperpigmentation of the skin and hypoglycemia symptoms were also common, in accordance with the study of Polat et al (23) who reported a large Triple A cohort (23 patients from 14 families) from Turkey. Therefore, symptoms of alacrima and achalasia must be investigated in all non-CAH PAI patients as the etiology may be TAS. Although Polat et al (23) reported short stature and PH in more than half of the cases, our cohort contained only one patient with short stature and PH was not present at all. Since our study has a retrospective design, PH may have been overlooked or not noted. On the other hand PH was only present in patients with the p.R478* mutation in the aforementioned study. This finding may be specific to the mutation. Grant et al (25) reported fissured palms in half of 20 patients in whom molecular analysis was not conducted. All of our patients with TAS had the homozygous p.L356Vfs*8 mutation in the *AAAS* gene, which was previously reported in a nine year old Turkish boy who had presented with achalasia, alacrima, stimulated cortisol deficiency, pitosis, pallor of optic disc, anisocoria and dry skin (26).

Familial glucocorticoid deficiency (FGD) is characterized by isolated GC deficiency in early infancy or in childhood, and presents with hyperpigmentation and hypoglycemia symptoms. All three of our patients were younger than two years at presentation and had a prolonged jaundice or history of it. In addition two of them presented with hyperpigmentation of skin and hypoglycemia. Akın et al (27) also reported the case of a 17-day-old newborn diagnosed with FGD type 1 who presented with hyperbilirubinemia and hyperpigmentation. Therefore, hyperpigmentation, persistent hypoglycemia and prolonged jaundice should suggest the possibility of AI in infancy. Although MC requirement and transient hyponatremia has been occasionally reported (16,28), none of our FGD patients had MC deficiency. Mutations in the *MC2R* gene (encoding the ACTH receptor protein) and *MRAP* gene (encoding MC2R accessory protein) are well described causes (almost 50%) of FGD (7). Although other rare genetic defects are also reported as causes in FGD, the underlying cause is unknown in about 40% of cases (2,7,29). These genetic defects manifest as phenotypically indistinguishable. Molecular genetic analysis of our patients revealed two different mutations in the *MC2R* gene, which have been previously reported (16,27,30).

Mutations in the *NR0B1 (DAX1)* gene located on Xp21.3-p21.2 and deletions in Xp21 (contiguous gene deletion) lead to impaired development of the adrenal glands, hypothalamus, pituitary gland and gonads and cause AHC. AI typically begins in early infancy or in childhood, but rarely begins in adulthood (31). Patients can also have hypogonadotropic hypogonadism (HH), which is characterized by undescended testes, micropenis, delayed puberty or infertility, associated with low levels of gonadotropins. It was reported that AHC due to *DAX1* mutation is a relatively frequent cause of non-CAH PAI in Chinese children (6,32). Wijaya et al (6) reported 20 male AHC cases (19 had *NR0B1* gene mutation) among 49 children with PAI. Of these, five patients presented with a typical adrenal crisis, 10 with salt craving, three with generalized hyperpigmentation at onset, and six patients with HH during follow-up. In this study the age at onset of AHC was <3 months in 13 of 20 patients, and ≤2 years in 17 of 20 patients. Lin et al (33) reported *DAX1* mutations in 58% (37 of 64) of 46,XY phenotypic boys with AI (not caused by CAH, ALD, or autoimmune disease) and in all boys (eight of eight) with HH and a family history suggestive of AI in males. AI had begun in early infancy in 81% of patients in their study. Only one male patient was diagnosed with AHC in our cohort, who had presented with salt wasting in the neonatal period. He had normal external genitalia and gonadotropin levels. Molecular analysis revealed a novel p.Q301* hemizygous non-sense mutation in the *DAX1* gene.

In recent years, many syndromic diseases that can cause PAI have been identified (Table 1). IMAGe syndrome was primarily defined by a spectrum of intrauterine growth restriction, metaphyseal dysplasia, CAH and genital anomalies. Patients can also have dysmorphic craniofacial features, hypocalcemia, and scoliosis (34). This disease is caused by gain of function mutations in the cyclin dependent kinase inhibitor (*CDKN1C*) gene, which regulates prenatal and postnatal growth. MIRAGE syndrome, another known cause of syndromic PAI, is due to a heterozygous *SAMD9* gain of function mutations and is characterized by myelodysplasia, infection, restriction of growth, adrenal hypoplasia, genital anomalies and enteropathy. Neurological findings, such as microcephaly, hydrocephalus, white matter abnormalities, and perivascular calcifications were also described (35). Our two patients with neonatal onset PAI had dysmorphic features. One of them (P19) had severe pre- and post-natal growth retardation, dysmorphic facial features and urogenital anomalies accompanying PAI and was clinically diagnosed as IMAGe syndrome. The other one (P22), in contrast to IMAGe and MIRAGE syndrome, had no growth retardation, so it was considered that the neurological findings of the patient could have been due to severe hypoglycemic episodes and electrolyte imbalances during the newborn period, or caused by a peroxisomal or undefined syndromic disorder.

According to both the literature and our study, in at least 80% of children with non-CAH PAI, the etiology can be determined by specific clinical and laboratory findings with or without molecular analysis of a candidate gene. For patients who do not exhibit specific clinical findings, predicting the exact etiology can be challenging. Nevertheless, establishing a specific genetic diagnosis in PAI is very valuable for a number of reasons: 1) providing clear information about disease spectrum, potential comorbidities and prognosis; 2) modifying treatments, such as requirement for MC replacement; 3) genetic counseling of affected individuals and their families, identifying presymptomatic children before onset of potentially life-threatening symptoms; and 4) increasing knowledge about the normal biology and pathomechanisms of PAI (2). Comprehensive diagnostic algorithms for PAI in children are available in the literature (5,7) For patients without a definite diagnosis despite referral to these algorithms, gene panel based next generation sequencing, whole exome sequencing, or array comparative genomic hybridization are now more widely available.

### Study Limitations

One of the limitations of our study was its retrospective design. Another limitation was that diagnostic molecular analysis could not be performed for all patients due to the limit in resources, especially in the earlier years of the study period.

## Conclusion

As PAI is a life-threatening condition, early recognition and proper treatment are crucial. Signs, such as hyperpigmentation of skin, recurrent hypoglycemic episodes with or without prolonged jaundice, chronic fatigue and hyponatremia with hyperkalemia could most likely suggest AI in children. During diagnostic studies, CAH must initially be excluded. Afterwards, specific clinical and laboratory features must be evaluated and proven with appropriate candidate gene analysis. Due to the many advantages, advanced molecular analysis should be considered for patients who have no specific diagnostic features.

## Figures and Tables

**Table 1:**
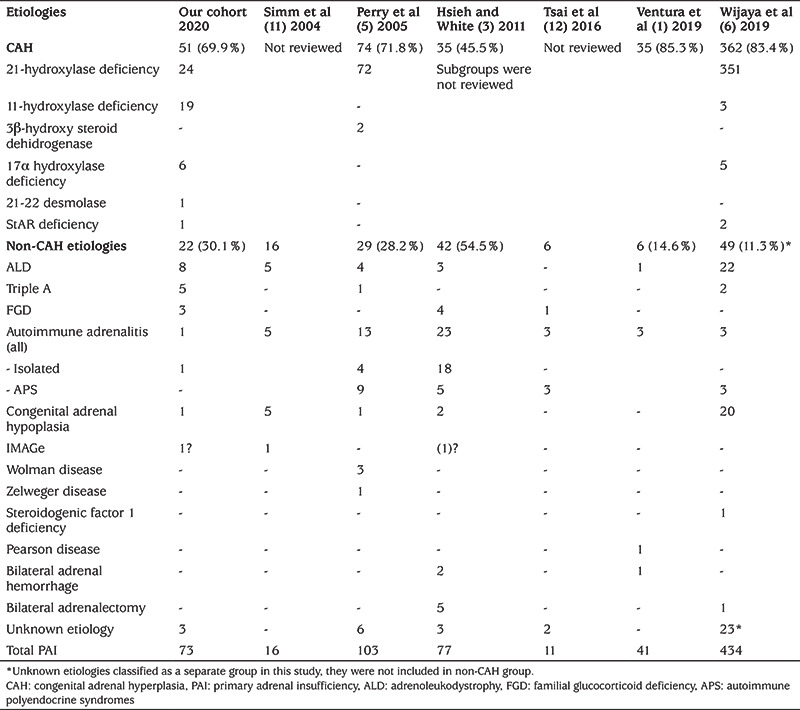
Etiologies of primary adrenal insufficiency in different cohorts

**Table 2:**
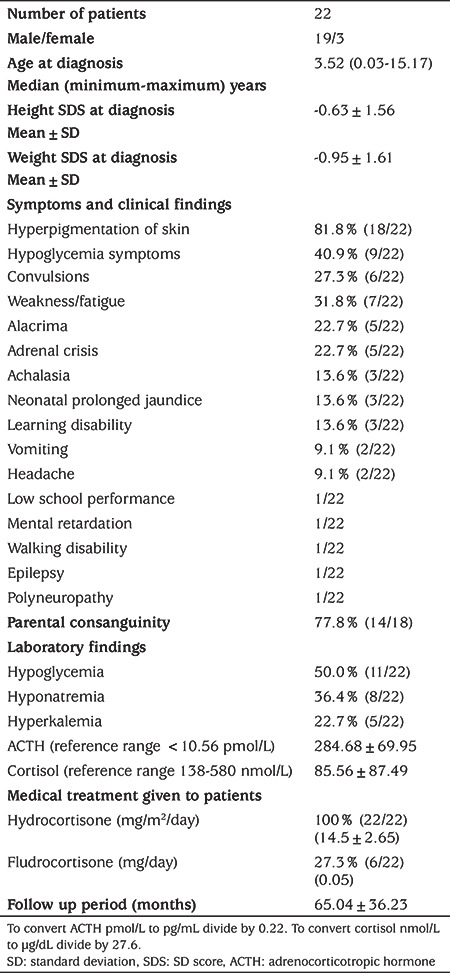
Non-congenital adrenal hyperplasia primary adrenal insufficiency patients’ characteristics, treatment and follow up

**Table 3:**
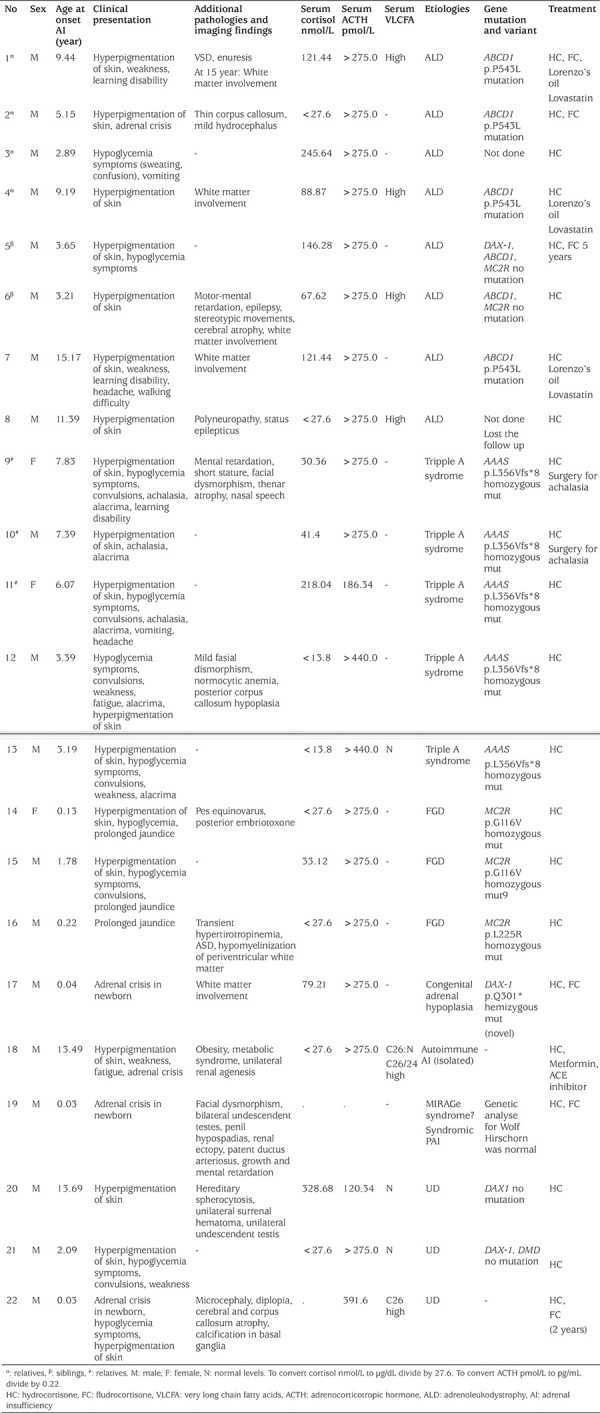
Etiologies and characteristics of non-congenital adrenal hyperplasia primary adrenal insufficiency patients
